# Neuropharmacological Insights into Glutamate Homeostasis in Post-stroke Depression Regulated by Astrocytes

**DOI:** 10.2174/011570159X379476250611052236

**Published:** 2025-06-30

**Authors:** Na Zhang, Kaijun Han, Lixinbei Sheng, Fang Wang, Manlan He, Mengqian Wu, Zhen Han, Yichen Li, Lu Chen

**Affiliations:** 1 Department of Nursing Research Institute, Nanjing Drum Tower Hospital, Affiliated Hospital of Medical School, Nanjing University, Nanjing, Jiangsu, China;; 2 Department of Neurosurgery, Nanjing Drum Tower Hospital, Affiliated Hospital of Medical School, Nanjing University, Nanjing, Jiangsu, China;; 3 Department of Thyroid Surgery, Nanjing Drum Tower Hospital, Affiliated Hospital of Medical School, Nanjing University, Nanjing, Jiangsu, China;; 4 Department of Pharmacy, Nanjing Drum Tower Hospital, Affiliated Hospital of Medical School, Nanjing University, Nanjing, Jiangsu, China

**Keywords:** Astrocytes, excitotoxicity, glutamate, glutamate transporter, glutamate receptor, neuropharmacology, post-stroke depression

## Abstract

Stroke patients often experience multiple functional impairments, including difficulties with swallowing, speech, cognition, and motor skills, which can lead to symptoms such as emotional distress and cognitive deficits. Approximately one-third of post-stroke patients may develop post-stroke depression (PSD), significantly hindering recovery and increasing the burden on families and healthcare systems. This review focuses on the underlying mechanisms of PSD, emphasizing the glutamatergic hypothesis. As the primary excitatory neurotransmitter, glutamate plays a central role in neural-signaling. However, excessive glutamate accumulation can cause neuronal damage, making it a key mechanism in the development of PSD. Astrocytes are crucial for maintaining glutamate homeostasis by clearing excess glutamate and regulating its synthesis and transport, thereby preventing excitotoxicity. Following a stroke, astrocytic dysfunction—characterized by overactivation and inflammatory responses—can exacerbate neuronal injury and further contribute to the emergence of depressive symptoms. This article also highlights potential therapeutic approaches targeting the glutamatergic system, such as NMDA receptor antagonists, AMPA receptor antagonists, and modulators of glutamate transporters, as well as other types (*e.g*., Chinese medicine, herbal medicine, and targeted pathways acting on neurons). These strategies offer promising avenues for PSD treatment. Future studies should delve deeper into the molecular mechanisms by which astrocytes regulate glutamate homeostasis, providing a robust foundation for the precision treatment of post-stroke depression.

## 1 INTRODUCTION

Stroke is one of the leading causes of disability and death among adults worldwide. Stroke patients often experience multiple functional disabilities during their recovery process, including swallowing, language, cognition, and motor function, which not only affect their daily living ability but also have a profound impact on their mental health [1]. Patients often experience tremendous physical and psychological pressure, which can lead to feelings of helplessness and depression and ultimately threaten their mental health and recovery process [2]. Among the brain stroke-related damages, emotional disorders have the most significant impact on a patient's survival and quality of life, with depressive symptoms being the most common [3]. Studies have shown that up to one-third of stroke survivors may develop post-stroke depression, which is a common emotional disorder after stroke and clinically characterized by symptoms, such as poor concentration, sadness, low mood, and irritability. Early symptoms are usually mild, but as the condition progresses, patients gradually develop symptoms of lethargy, tension, weight loss, and even suicidal tendencies [4]. Due to physical, cognitive, language, and emotional impairments, patients' social interactions decrease, social isolation worsens, and the desire to rejoin society decreases. The occurrence of this emotional disorder not only significantly affects patients' recovery progress but also increases the burden on families and healthcare systems [2, 5-7].

The pathogenesis of PSD is complex, with social, psychological, and biological mechanisms all playing a role in disrupting the physiological and psychological balance of stroke patients. The causes of PSD are subject to debate due to the influence of different research methods and sample sizes. Evidence suggests that neurobiological factors are the leading cause, including abnormal nutritional responses, the hypothalamic-pituitary-adrenal axis (HPA) axis dysfunction, glutamate-mediated neurotoxicity, and imbalances in neurotransmitters and neuroinflammation [8]. Neurotrophic factor imbalances, neuroinflammation, and hormonal abnormalities may all play a crucial role in developing PSD [7, 9, 10]. Following a stroke, as a compensatory and adaptive response to neural tissue damage, neurotrophic activity, *i.e*., neuron growth and maturation, increases dramatically, and neurotrophic factors exert neuroprotective effects and promote neural repair after injury [11]. Research has found that PSD patients have lower serum levels of brain-derived neurotrophic factors than those without depressive symptoms [12]. A deficiency of brain-derived neurotrophic factors may increase the risk of severe depression and PSD and affect treatment outcomes [11]. Following a stroke, central and peripheral pro-inflammatory cytokine levels rise, inducing inflammation and leading to HPA axis dysfunction. Both inflammation and HPA dysfunction can increase the risk of PSD through various mechanisms that reduce the transcription of neurotrophic factors [7, 13].

The research on the pathogenesis of post-stroke depression, especially the glutaminergic hypothesis, has garnered attention. Glutamate, as the main excitatory neurotransmitter in the central nervous system, is involved in learning, memory, cognition, and other vital functions, and its abnormal regulation is considered one of the potential pathological mechanisms of post-stroke depression [14]. After stroke, brain injury leads to abnormal neuronal function, and imbalance of glutamate synthesis, transport, and receptor regulation may lead to excessive accumulation of glutamate, which may lead to excitatory toxicity and neuronal injury. This process involves the dysfunction of glutamate transporters and receptors and is closely related to the abnormal activation of astrocytes. Astrocytes play an important role in maintaining glutamate homeostasis in the brain, and their dysfunction may aggravate nerve damage after stroke and further promote the occurrence of depressive symptoms [10, 15].

In recent years, there has been an increasing number of studies on how to improve psychological health and enhance the ability to adapt to life of stroke survivors, especially in terms of treatment strategies for PSD. Interventions based on the glutamate system, especially the study of drugs targeting glutamate transporters and receptors, have become a research hotspot [16, 17]. These studies provide new theoretical support for the clinical treatment of post-stroke depression and also point to potential drug targets for future drug development.

## 2 GLUTAMATE SYNTHESIS, TRANSPORT, AND EXCITATORY CONDUCTION IN THE CNS

### 2.1 Cellular Glutamate Synthesis and Transport

#### 2.1.1 Glutamate Synthesis

Glutamate is the primary excitatory neurotransmitter in the nervous system. It is widely present in the cerebral cortex, hippocampus, cerebellum *etc*. participating in various cognitive, motor, and sensory functions, mediating excitatory synaptic transmission, and serving as a metabolic hub involved in glucose and amino acid metabolism [18]. 90% of synapses in the forebrain are glutamatergic, and excitatory transmission is mediated by the exocytosis of vesicles containing glutamate from the presynaptic neuron, binding to receptors on the postsynaptic membrane and activating them, thereby mediating excitatory transmission [19]. In the nervous system, due to the blood-brain barrier, glutamate in the peripheral blood cannot enter the nervous system [20]. Therefore, glutamate mainly relies on glucose metabolism and the glutamate-glutamine cycle to synthesize.

Glucose is an essential substrate for the synthesis of glutamate [21], which can be synthesized from α-ketoglutarate (α-KG), an intermediate of the tricarboxylic acid (TCA) cycle, through glycolysis and the TCA cycle, in both neurons and astrocytes [22]. After glucose is converted to pyruvate (Pyr) in the cytosol of neurons and astrocytes through glycolysis, Pyr can enter the TCA cycle and generate α-KG in two ways. On the one hand, pyruvate can enter the TCA cycle through pyruvate dehydrogenase (PDH), generating α-KG [23]. On the other hand, it can be carboxylated by pyruvate carboxylase (PC) to generate oxaloacetate (OAA) and enter the TCA cycle, generating downstream intermediates α-KG [24, 25]. However, it is worth noting that PC mainly exists in the mitochondria, so this process primarily occurs in astrocytes [25]. α-KG and amino are synthesized into glutamate by glutamate dehydrogenase (GDH) under the catalytic action of GDH, which is reversible [26]. Synthesized glutamate is released from the presynaptic neuron into the synaptic cleft and mediates the excitability of the postsynaptic neuron.

Another way to synthesize glutamate is the glutamate-glutamine cycle, a highly active cyclic process between glutamate neurons and astrocytes in the synapse [27]. Among all the cells in the nervous system, astrocytes are closely related to glutamate synapses and are responsible for absorbing most of the glutamate in the synaptic cleft [28]. In contrast, neurons absorb only a tiny part of it. Afterabsorption into astrocytes, glutamate is converted into glutamine by glutamine synthetase (GS) and then released from astrocytes, which is assisted by glutamine transporters (mainly Sodium-coupled Neutral Amino acid Transporters, SNATs) expressed in both neurons and astrocytes [29]. The exocytosis of SNATs in astrocytes is related to additional H^+^ exchange [30], which does not exist in primary neurons, so glutamine is taken up by SNATs in neurons and then phosphorylated by phosphate-activated glutaminase (PAG) to form glutamate [31]. Excitatory amino acid transporter (EAAT) is the leading glutamate uptake transporter in the CNS [32]. The glutamate produced by neurons is released into the intercellular space as vesicles and then transported into astrocytes by EAAT, which is converted back into glutamine by GS again to form the cycle [33].

#### 2.1.2 Glutamate Transport

Glutamate is mainly transported in the nervous system through transporters. Five glutamate transporters have been identified, including the glutamate/aspartate transporter (GLAST/EAAT1), glutamate transporter 1 (GLT-1/EAAT2), excitatory amino acid transporter 1 (EAAC1/EAAT3), excitatory amino acid transporter 4 (EAAT4), and excitatory amino acid transporter 5 (EAAT5) [34]. GLAST and GLT-1 are mainly expressed in astrocytes [35]. GLAST is a membrane-bound transporter that co-transports glutamate, three Na^+^, and one H^+^ against the concentration gradient and transports one K^+^ in the opposite direction [36]. When GLAST is inhibited, the extracellular glutamate level increases, leading to excitotoxic death of neurons [37]. GLT-1 is a Na^+^-dependent transporter across the membrane and a significant subtype for clearing inter-synaptic glutamate [38, 39]. EAAC1 is mainly expressed in neurons, especially on the postsynaptic membrane, and controls glutamate-dependent neural transmission and synaptic plasticity [40]. Other subtypes, such as EAAT4, are expressed primarily in the cerebellar Purkinje cells [41], and EAAT5 is mainly expressed in retinal photoreceptor cells [42]. These subtypes play essential roles in glutamate transmission in specific brain regions. The process is detailed in Fig. (**1**).

### 2.2 Glutamate Receptors And Excitatory Transmission

Glutamate is released into the synaptic cleft, where it primarily acts on two major classes of receptors on the postsynaptic membrane: ionotropic glutamate receptors (iGluRs) and metabotropic glutamate receptors (mGluRs). Ionotropic glutamate receptors are divided into NMDA (N-methyl-D-aspartate), AMPA (α-amino-3-hydroxy-5-methyl-4-isoxazolepropionic acid, α-amino-3-hydroxy-5-methyl-4-isoxazolepropionic acid), and KA (Kainate) receptors [43]. iGluRs are the most representative class of receptors in ligand-gated channels and have an extracellular glutamate binding site and a transmembrane ion channel that can directly regulate ion channels on the cell membrane to mediate excitatory transmission between neurons. These receptors are concentrated at the synapses of neurons. The ion channel opens when glutamate binds to the receptors, allowing Na^+^ and Ca^2+^ to flow into the cell, causing neuronal excitation [44]. mGluRs mainly regulate intracellular signaling by activating G protein signaling pathways. They mostly complete synaptic signal transmission by sensing glutamate levels in the synaptic cleft or the surrounding environment of neurons, thereby regulating neurotransmitter release, neuron growth *etc*. They are divided into three subtypes: I, II, and III [45].

## 3 DISORDER OF THE GLUTAMATE SYSTEM IN PATIENTS WITH PSD

Glutamate is the primary excitatory neurotransmitter in the CNS, and it regulates the excitability of neurons by binding to specific receptors in normal physiological conditions. However, PSD is accompanied by changes in glutamate levels in the frontal lobe, and plasma glutamate levels at admission are closely related to the development of PSD within 3 months [46, 47]. In the PSD astrocyte group, the density of glutamate in CNS-derived astrocytes is not consistent with the trend of glutamine changes, indicating a disorder in the glutamate cycle in CNS-derived astrocytes. The main mechanisms include the following aspects.

### 3.1 Excessive Release and Accumulation of Glutamate

During a stroke, the concentration of glutamate in cerebrospinal fluid (CSF) and extracellular fluid increases by more than 300 times, and the rise in glutamate may disrupt the blood supply to dead tissue in the brain (ischemia), leading to large-area infarction and further promoting the diffusion of glutamate, causing neuronal damage in areas outside the infarcted tissue. Overstimulation of glutamate receptors by excess glutamate leads to cell swelling, cell apoptosis, and neuronal death, resulting in poor neurological prognosis [48]. This excess glutamate is overactivated not only ionotropic glutamate receptors on the postsynaptic membrane, such as NMDA receptors and AMPA receptors [49], but also activates mGluRs [50], leading to a massive influx of Ca^2+^ and ultimately causing neuronal death.

### 3.2 Glutamate-induced Excitotoxicity

Excitatory toxicity induced by glutamate is one of the main mechanisms of neuronal injury in PSD. Under normal circumstances, glutamate transporters (such as GLT-1) are responsible for clearing glutamate from the synaptic clefts and maintaining homeostasis. However, cerebral ischemia can impair these transporters' function, reducing the efficiency of glutamate clearance and leading to its accumulation outside the cell, further exacerbating excitotoxicity. At the same time, when glutamate receptors are activated in large quantities, it causes an imbalance in the brain, increasing the production of free radicals to toxic levels. Free radicals consist of atoms and molecules that can exist independently and are often highly reactive, so much so that they can oxidize and damage proteins [51]. In particular, there is an excessive accumulation of Ca^2+^; when glutamate receptors are over-stimulated, ATP is depleted, and ion homeostasis is interrupted. During the activity, the influx of sodium and calcium into the cell affects all subcellular compartments, including the cytosol, endoplasmic reticulum (ER), nucleus, and mitochondria, leading to significant changes [52]. It also activates various degradation enzymes, such as calpain and phospholipase, and promotes the generation of reactive oxygen species (ROS), exacerbating cell damage [17, 53]. When glutamate and calcium metabolism are disrupted, glutamate transporter proteins are damaged, and glutamate receptors (AMPA-alginate and NMDA) are impaired; glutamate receptor-induced damage to cells, especially when the brain is under stress, can trigger excitotoxicity [19], which plays a central role in PSD.

On the other hand, the excitatory amino acid transporter system is also damaged, so it cannot remove excess glutamate from the synaptic cleft. Severe neurological dysfunction caused by widespread infarction can significantly damage important regions that regulate emotional behavior and biochemical changes in the brain [54]. The loss of neurons in the localized areas of the brain can disrupt the transmission of sensory input, including emotional and emotional regulation [55], induce brief depolarization and epileptiform discharges in CA1 pyramidal neurons in the hippocampus, leading to dysregulation of neuropsychiatric disorders [56].

## 4 REGULATION OF GLUTAMATE HOMEOSTASIS BY ASTROCYTES IN PATIENTS WITH PSD

### 4.1 Overview of the Physiological and Pathological Mechanisms of Astrocytes in the CNS

Astrocytes are diverse and complex glial cells that play essential roles in maintaining the CNS's (CNS) stability and supporting neurons in neural networks. They are widely distributed throughout the CNS and have functions such as regulating synapse formation, modulating neurotransmitter transmission, and maintaining ion balance. Astrocytes also have a variety of physiological functions, including the clearance of neurotransmitters and the promotion of synaptic plasticity and neural network remodeling. Additionally, astrocytes regulate the formation and stability of the blood-brain barrier through their perivascular end feet and provide metabolic support to neurons, such as lactate delivery, to meet the high energy demands of neurons [57]. In conditions of neural injury and disease, astrocytes can be activated to release cytokines and growth factors and play a neuroprotective role. However, they may mediate inflammatory responses and affect disease progression [58]. Recently, the role of astrocytes in neural development, synapse formation, and neurodegenerative diseases has received widespread attention, and their potential as a therapeutic target is being explored [59]. These characteristics suggest that astrocytes play an indispensable role in maintaining the stability of the CNS and regulating disease progression.

### 4.2 Regulation of Glutamate Homeostasis by Astrocytes in Patients With PSD

As shown in Fig. (**2**), astrocytes play a role in balancing the secretion and uptake of glutamate. The precise regulation of glutamate concentration in synaptic networks controls the network's excitability, plasticity, and synchronized activity. Glutamate and D-serine accumulate in synaptic-like microvesicles (SLMVs) around astrocytic synapses, and astrocytes regulate glutamate exocytosis by rapidly releasing glutamate [60]. After glutamate receptor activation, astrocytic vesicles undergo rapid (millisecond-level) Ca^2+^ and SNARE-dependent exocytosis, accompanied by glutamate release [61]. Astrocytes rapidly clear excess glutamate from the synaptic cleft by high expression of glutamate transporters such as GLT-1 and GLAST [62].

Additionally, these cells convert glutamate into glutamine through glutamate synthetase, which can be re-absorbed by neurons and converted back into glutamate, achieving neurotransmitter recycling. The metabolic activity of astrocytes forms a “glutamate-glutamine cycle” with neurons [63]. Recent research published in the journal Nature shows that astrocytes targeted deletion of vesicular glutamate transporter 1 (VGLUT1) selectively expressed to inhibit glutamate release, thereby effectively limiting glutamate concentration in the nervous system, which is vital for maintaining neurotransmitter supply and preventing excitotoxicity.

After a stroke, astrocytes show significant morphological changes, characterized by enlarged cell size and thicker processes. Additionally, astrocytes often redistribute in the damaged area, forming a barrier around damaged blood vessels and neurons to limit damage spread, which can have beneficial and harmful effects [64]. After a stroke, astrocytes significantly upregulate inflammation-related molecules such as tumor necrosis factor-α (TNF-α), interleukin-1β (IL-1β), and matrix metalloproteinases (MMPs) to regulate inflammatory responses and extracellular matrix remodeling, which affect the progression of brain injury [65]. At the same time, they release neurotrophic factors, including brain-derived neurotrophic factor (BDNF) and glial cell line-derived neurotrophic factor (GDNF), which promote neuron survival and axon regeneration and have potential neuroprotective effects [66]. In excitotoxicity caused by a stroke, astrocytes regulate the expression of GLT-1 and GLAST to effectively remove excess glutamate around synapses, thereby alleviating neurotoxicity [67]. However, excessive activation of astrocytes in the later stage of a stroke can lead to glial scar formation. This scar isolates inflammatory regions while impeding axon regeneration, negatively affecting neural function recovery [68]. The release of large amounts of pro-inflammatory factors by astrocytes after a stroke may amplify the inflammatory response and exacerbate neuron injury [69].

## 5 INTERVENTIONS TO MODULATE GLUTAMATE HOMEOSTAT IN PSD REGULATED BY ASTROCYTES

The mechanism of PSD is similar to that of other depressive disorders. However, it is still difficult to control and often leads to treatment failure, which has aroused widespread academic attention. In the past, based on the monoamine theory, the Food and Drug Administration (FDA) approved the primary drugs used to treat PSD, including tricyclic antidepressants, selective serotonin reuptake inhibitors (SSRIs) [70], and serotonin-norepinephrine reuptake inhibitors (SNRI) [71]. In recent years, the treatment of depression has focused on the role of the glutamate system, which is expected to provide future drug treatment for PSD patients.

### 5.1 Ioic Receptor Antagonists

Many clinical studies have revealed the antidepressant effects of drugs that antagonize NMDA receptors, leading to the development of drugs that target the same receptors, Including NR2B selective compound (Ro25-6981) [72], NR2B selective subunit (CP-101,606) [73], dextromethorphan/bupropion [8], Memant [74], magnesium [17], NR2B antagonist (MK-0657) [75], AZD6765 [76], trisodil [77], NRX1047, GLYX-13, D-cycloserine [78], zinc [79], MK-801 [80] and CGP37849 [81].

At the same time, antagonists are targeting the alpha-amino-3-hydroxy-5-methyl-4 isoxazolinic acid (AMPA) receptor. At least six targeted drugs are known to be potential treatments for depression: aniracetam [82], piracetam [83], amprapine, CX614 [84], LY392098 [85], and LY451646 [86]. Recently, the US Food and Drug Administration approved the NMDA receptor and AMPA receptor antagonist Esketamine for the treatment of patients with drug-resistant depression [87, 88].

### 5.2 Metabolic Receptor Antagonists

Recent studies have shown that metabolic receptor antagonists, mainly include: mGluR1 [89] and mGluR5 [90] (group I) have antidepressant effects; mGluR2 and mGluR3 (Group II) selective agonists (R,S)-4-carboxyl-3-hydroxyphenylglycine (CHPG), (2S,1'R,2'R,3'R)-2-(2,3-dicarboxycyclopropyl) glycine (DCG-IV) and (2S, 3'R) 3S, 4S)-alpha-(Carboxycyclopropyl) glycine (L-CCG-I) inhibited excitation conduction [91]. There are also mglur4 [92], mglur7 [93], mglur6, and mGluR8 [94] (Group III).

### 5.3 Glutamate Transporters

The antidepressant activity has been shown to result not only from drugs that modulate glutamatergic synapses but also from those that modify the glutamate transporter proteins responsible for the cellular uptake of glutamate. There is evidence that GLT-1 inhibitor DHK reduces the function of glutamate transporter 1 [95]; fluoxetine (FLX) treatment reverses behavioral deficits and chronic unpredictable stress-induced decreases in GLT-1 levels [96]; and the use of mesenchymal stem cell-EAAT therapy can also improve depressive-like symptoms [97].

### 5.4 Glutamate Homeostasis Regulated By Astrocytes

Harmine is a natural β-carboline alkaloid and an effective psychoactive substance known to regulate astrocytes' glutamate transporter. Harmine may play a similar role in antidepressants by restoring astrocyte function [98]. TNF-α activates CXCR4 to release glutamate from astrocytes, leading to the amplification effect of microglia and causing neurotoxicity. The change in astrocyte communication will directly lead to neuropathological consequences, and drugs that interfere with CXCR4-mediated signaling between astrocytes and microglia can prevent HIV-1 envelope glycoprotein gp120IIIB-induced neuronal apoptosis [99]. O-GlcNAc transferase (OGT) in astrocytes regulates depressive-like behavior by regulating GLT-1 O-GlcNAcylation, and specific knockout may be a potential target for antidepressants [100]. Other glutamate-based drugs have also similar antidepressant effects, including minocycline and riluzole. DMT is an essential amino acid metabolic pathway that can cause activation of the NMDA receptor in the brain [100]. Riluzole is an NMDA, AMPA, and kainate receptor antagonist that can prevent presynaptic glutamate release and promote the uptake of relatively high concentrations of glial glutamate [101].

### 5.5 Others

SF-11 is a Y2R antagonist that can induce a rapid antidepressant-like response, which may be related to its ability to inhibit glutamate neurotransmitters [102]. Glutamate cysteine-γ-lyase (GOT) clears glutamate from the brain by degrading glutamate through blood glutamate metabolism, and it has been proposed that this enzyme could be an effective new anti-depressive tool for treating depression in ischemic stroke patients [17]. JDTLG can regulate the NMDAR/ BDNF pathway to lower glutamate levels and increase γ-aminobutyric acid (GABA) levels, which may be essential in developing PSD [103]. Electroacupuncture (TNEA) upregulated the expression of post-synaptic cell adhesion molecule Netrin-G ligand-3 (NGL-3) in the mPFC. Further research showed that the extracellular domain of NGL-3 binds to the presynaptic protein L1cam, promoting the formation of PSD treatment-effective molecular and synaptic mechanisms and emphasizing the potential of targeting the NGL-3/ L1cam pathway for developing alternative interventions for PSD and other depressions [104]. The primary neuronal transcription factor NeuroD1 treatment significantly improved motor, sensory-motor, and psychological function in stroke patients post-stroke [105]. Serum transport inhibitors can treat PSD [106]. Other drugs that have been proven effective are norepinephrine and dopamine reuptake inhibitors (NDRIs) and monoamine oxidase inhibitors (MAOIs) [107].

## 6 DISCUSSION AND PROSPECTS

This review highlights the crucial role of astrocytes in regulating glutamate homeostasis in patients with post-stroke depression, providing a new perspective on the pathogenesis of PSD). The study indicates that the excessive release of glutamate and the imbalance in its transport mechanism are the main pathogenic mechanisms of PSD, and astrocytes play a central role in maintaining glutamate homeostasis. Specifically, glutamate transporters (GLT-1 and GLAST) clear excessive glutamate from synaptic clefts rapidly, effectively alleviating excitotoxicity. Additionally, astrocytes support the recycling of synaptic glutamate through the glutamine cycle, guaranteeing neurotransmitter homeostasis. However, dysfunction of astrocytes after stroke, including excessive activation leading to inflammation and formation of glial scar, may exacerbate neurotoxic injury.

Theoretically, this review further enriches the glutamatergic hypothesis of PSD, emphasizing that astrocytes are not only supportive cells but also essential participants in disease regulation. Practical intervention strategies targeting the glutamate system, including receptor antagonists, transporter modulators, and drugs based on astrocyte function, show promising therapeutic prospects. These findings provide a clear direction for future research on the precision treatment of PSD. Nevertheless, the double-edged sword effect of astrocytes remains an important challenge in future treatment design. On the one hand, it exerts positive effects on stroke recovery by releasing neurotrophic factors and regulating neural network remodeling; on the other hand, excessive activation may trigger inflammation and tissue scarring. Therefore, exploring intervention methods must consider a dynamic balance between their protective effects and potential damage. Precision treatment methods targeting specific brain regions still need further optimization since the glutamate system involves extensive neural regulation functions.

In the future, the molecular mechanism of astrocytes in maintaining glutamate homeostasis and treating related diseases needs to be further explored. Through single-cell RNA sequencing and advanced imaging technology, the functional differences of astrocytes in specific brain subtypes are studied, which is expected to clarify further their molecular mechanisms in glutamate homeostasis and neural function regulation and help identify more precise therapeutic targets. Novel drug development: Research on drugs targeting the glutamate system (such as receptor antagonists and transporter modulators) will continue to expand, especially the development of specific regulators acting on astrocytes, for example, through the regulation of key transporters such as GLT-1 to intervene in neurotoxicity and neuronal damage. Interdisciplinary treatment strategies: Combining neuroscience and bioengineering, regulating astrocyte function through gene editing technology, or using stem cell therapy and biomaterials to repair brain tissue provide new interventions for neurological diseases. Synergistic studies with other cell types: In-depth exploration of astrocytes' interactions with neurons, microglia, and the vascular system, especially the dynamic changes in the pathological environment, will help to understand the complexity of the disease process.

## 7 LIMITATIONS

This article has several limitations. Firstly, it focuses on the regulation of glutamate and the role of astrocytes without a comprehensive discussion on other possible factors such as genes and social psychological environment. Secondly, the intervention measures proposed in this paper are primarily theoretical suggestions, with insufficient debate on the feasibility of specific implementation strategies and clinical applications. There is also a lack of comparative analysis on the effectiveness of existing intervention measures. At the same time, the universality of glutamate system dysfunction and astrocyte function disorder still needs more research to verify. Finally, future research needs further to strengthen the exploration of mechanisms and experimental verification and combine multidisciplinary perspectives to improve the theory's practical utility and clinical guidance value.

## CONCLUSION

PSD is a common post-stroke emotional disorder in clinical practice, and improving the psychological health of stroke patients and enhancing their ability to adapt to life are the key issues that scholars need to focus on in this field. The glutamate hypothesis of PSD pathogenesis has been widely discussed in recent years. Glutamate is the primary excitatory neurotransmitter in the nervous system, and its synthesis, circulation, and transportation is a highly active circulation process, which mainly depends on the glutamate-glutamine cycle between the presynaptic glutamate neurons and astrocytes. However, the glutamate system is disrupted in patients with post-stroke depression, leading to excessive release and accumulation of glutamate, excitotoxicity, and ultimately, cell swelling, cell apoptosis, and neuronal death. Astrocytes balance glutamate secretion and uptake, effectively limiting glutamate concentration in the nervous system, which is particularly important for maintaining neurotransmitter supply and preventing excitotoxicity. In this regulation process, various receptors and transporters are involved, and this article focuses on discussing the potential targets for developing post-stroke antidepressant drugs. For future research directions, studying the functional differences of astrocyte subtypes using single-cell RNA sequencing and integrating neuroscience and biological engineering will help further understand the disease progression and seek new treatment strategies.

## Figures and Tables

**Fig. (1):**
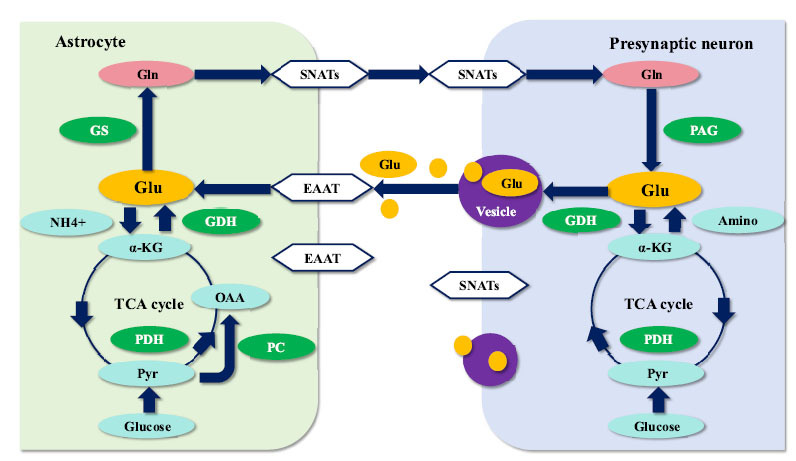
Synthesis, circulation, and transportation of glutamate. **Abbreviations**: α-KG: α-ketoglutarate; EAAT: Excitatory amino acid transporter; GDH: Glutamate dehydrogenase; Gln: Glutamine; Glu: Glutamate; GS: Glutamine synthetase; OAA: Oxaloacetate; PAG: Phosphate-activated glutaminase; PC: Pyruvate carboxylase; PDH: Pyruvate dehydrogenase; Pyr: Pyruvate; SNAT: Sodium-coupled neutral amino acid transporter; TCA cycle: Tricarboxylic acid cycle.

**Fig. (2):**
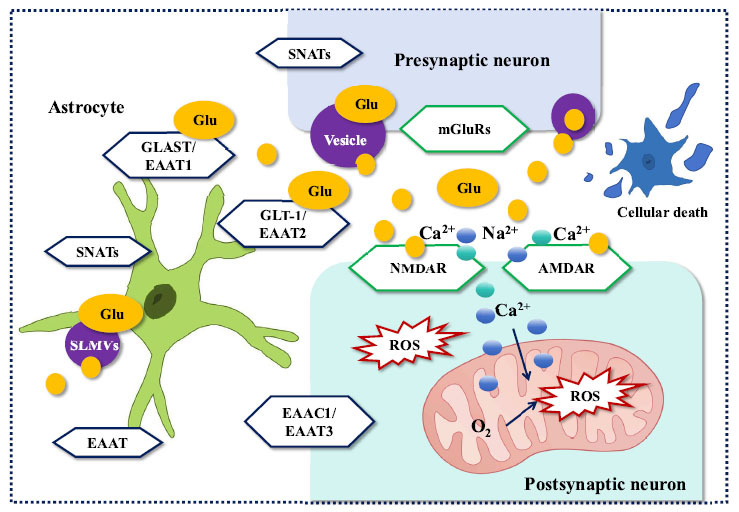
Regulation of glutamate homeostasis by astrocytes in patients with PSD. **Abbreviations**: AMPAR: α-amino-3-hydroxy-5-methyl-4-isoxazole propionic acid receptor; EAAC 1: Excitatory Amino Acid Carrier 1; EAAT: Excitatory amino acid transporter; GLAST: Glutamate/Aspartate Transporter; Glu: Glutamic acid; GLT-1: Glutamate transporter 1; mGluRs: Metabotropic glutamate receptors; NMDAR: N-methyl-d-aspartate receptor; ROS: Reactive oxygen species; SLMVs: synaptic-like microvesicles; SNAT: Sodium-coupled neutral amino acid transporter.

## LIST OF ABBREVIATIONS

BDNF: Brain-derived Neurotrophic Factor
CSF: Cerebrospinal Fluid
EAAT: Excitatory Amino Acid Transporter
GDH: Glutamate Dehydrogenase
GS: Glutamine Synthetase
HPA: Hypothalamic-pituitary-adrenal Axis
IL-1β: Interleukin-1β
PAG: Phosphate-activated Glutaminase
PC: Pyruvate Carboxylase
PSD: Post-stroke Depression
ROS: Reactive Oxygen Species
SLMVs: Synaptic-like Microvesicles
SNRI: Serotonin-norepinephrine Reuptake Inhibitors
SSRIs: Selective Serotonin Reuptake Inhibitors
TCA: Tricarboxylic Acid
TNF-α: Tumor Necrosis Factor-α
α-KG: α-ketoglutarate
